# Anatomy of the Episodic Buffer: A Voxel-Based Morphometry Study in Patients with Dementia

**DOI:** 10.1155/2008/828937

**Published:** 2008-04-11

**Authors:** M. Berlingeri, G. Bottini, S. Basilico, G. Silani, G. Zanardi, M. Sberna, N. Colombo, R. Sterzi, G. Scialfa, E. Paulesu

**Affiliations:** ^1^Psychology DepartmentUniversity of Milano-BicoccaMilanoItaly; ^2^Cognitive Neuropsychology LaboratoryNiguarda Ca' Granda HospitalMilanoItaly; ^3^Psychology DepartmentUniversity of PaviaPaviaItaly; ^4^Clinical Neuroscience DepartmentNiguarda Ca' Granda HospitalMilanoItaly; ^5^Neuroradiology DepartmentNiguarda Ca' Granda HospitalMilanoItaly

**Keywords:** Alzheimer's disease, immediate prose recall, episodic buffer, memory encoding, voxel-based morphometry, working memory

## Abstract

In 2000 Baddeley proposed the existence of a new component of working memory, the episodic buffer, which should contribute to the on-line maintenance of integrated memory traces. The author assumed that this component should be critical for immediate recall of a short story that exceeds the capacity of the phonological store. Accordingly, patients with Alzheimer’s dementia (AD) should suffer of a deficit of the episodic buffer when immediate recall of a short story is impossible. On the other hand, the episodic buffer should be somewhat preserved in such patients when some IR can occur (Baddeley and Wilson, 2002). We adopted this logic for a voxel-based morphometry study. We compared the distribution of grey-matter density of two such groups of AD patients with a group of age-matched controls. We found that both AD groups had a significant atrophy of the left mid-hippocampus; on the other hand, the anterior part of the hippocampus was significantly more atrophic in patients who were also impaired on the immediate prose recall task. Six out of ten patients with no immediate recall were spared at “central executive” tasks. Taken together our findings suggest that the left anterior hippocampus contributes to the episodic buffer of the revised working memory model. We also suggest that the episodic buffer is somewhat independent from the central executive component of working memory.

*List of abbreviations:* AD = Alzheimer disease; cRPM = Raven Coloured Progressive Matrices; DR = Delayed recall; EB = Episodic buffer; FDR = False Discovery Rate correction; FWE = Family–Wise Error correction; G-AD = Global ADs; GM = Grey matter; IR = Immediate recall; LTM = Long-term memory; MMSE = Mini Mental State Examination; S-AD = Selective ADs; VBM = Voxel-based morphometry; WM = Working memory.

## 1. Introduction

### 1.1. The new component of working memory

Baddeley [1] recently proposed the existence of a new component of working memory (WM) that he called *episodic buffer* (EB): a limited-capacity temporary storage system that should contribute to the on-line

ISSN 0953-4180/08/$17.00 © 2008 – IOS Press and the authors. All rights reserved maintenance of integrated mental images made of multiple elementary attributes, such as those of episodic memory, controlled by the central executive and connected with episodic long-term memory (LTM).

To assess whether the functions of the EB could be distinguished from those of LTM storage, Baddeley and colleagues [2] tested immediate and delayed recall of a short story that exceeded the capacity of a short-term memory span in patients with amnesia and patients with dementia of the Alzheimer type (AD). They found a variable degree of immediate recall (IR) impairment, which correlated with deficits of executive functions and concluded that IR reflected both the capacity of the EB and the efficiency of the central executive to support the creation and the maintenance of complex mental representations.

However, a recent study by Gooding et al. [3] failed to replicate these results with another group of amnesic patients in that IR recall was not correlated with executive functions: they explained this discrepancy by postulating a long-term memory contribution to immediate prose recall in their sample.

### *1.2. Anatomical evidence on the episodic buffer*

In the fMRI literature there is some evidence that suggests the right frontal lobe as a specific anatomical substrate for the EB [4–6]. However, it remains to be established whether this is the only anatomical correlatex: indeed, the paradigms used in the fMRI studies are substantially different from those adopted with neuropsychological patients such as patients with Alzheimer's disease, a disorder characterised by widespread brain abnormalities involving other systems [7,8].

### *1.3. Aims of the study*

Despite its theoretical interest, the novel concept of an EB of working memory requires to be better specified at the psychological and at the neuropsychological level. For example, its relationship with the central executive proper [2] and its neural substrates are still unclear. Indeed, the neural correlates of the EB have been investigated only in normal subjects and with tasks that were very different from those that were originally used in patients [4–6].

In this study we further explored these issues by comparing the neuropsychological profile and the neuroanatomical patterns of two groups of patients with mild dementia and a behavioural profile compatible with a different involvement of the EB. The anatomical patterns of these patients and the correlations with their behaviour were explored through the MRI voxel-based morphometry technique (VBM) [9,10].

## 2. Materials and methods

### *2.1. Subjects and neuropsychological testing*

Twenty-one AD patients complying with the NINCDS-ARDA criteria [11], and twenty-two normal controls participated in the study (see Table 1 for demographic variables).

Participants underwent to a neuropsychological battery which assessed general cognitive functioning and the main cognitive functions (see Table 1).

The AD-patient sample was divided in two groups on the basis of their performance in the short-story memory task. Ten out of the twenty-one patients showed some sparing when asked to recall a short story immediately after the first presentation (immediate recall – IR task). On the other hand, 10 minutes after a further presentation of the story (delayed recall – DR task) none of the AD patients could recall any element of the story. Patients with relative sparing of IR are hereafter called "selective ADs" (S-AD); patients with a memory deficit for both IR and DR are called "global ADs" (G-AD).

Performance in the short-story test was scored by counting the number of "idea units" or single elements recalled by the subjects, as in Baddeley's study [2].

Comparison of performance in the neuropsychological tests was calculated through the Mann-Whitney U tests; a correction for multiple comparisons was used (p-value threshold: 0.0015).

### *2.2. MRI Anatomical methods*

Magnetic Resonance Imaging was performed on a 1.5 Tesla Marconi Philips Infinion. A high-resolution, T1-weighted anatomical scan was acquired for each subject using a MPRAGE sequence (flip angle 35°, TE = 5 ms, TR = 21 ms, FOV = 256 × 192 mm, matrix 256 × 256, TI = 768 ms), thus obtaining 132 axial slices with 1x1x1 mm voxels. Data were analysed using Statistical Parametric Mapping (SPM 2, Wellcome Department of Imaging Neuroscience, London, UK, 2000) with a optimised VBM protocol [10]. The anatomical differences between the three groups were estimated with a t-test analysis on a voxel-by-voxel basis. We computed the following effects: a) areas of shared atrophy in the two groups of patients versus normal controls; b) areas of grey matter (GM) density loss in the G-AD group versus the controls and S-AD. In all the VBM analyses age and education were covaried out, as age (for the S-Ads) and education (for the G-Ads) were significantly different in comparison with controls (Mann-Whitney U test: $p < 0.008$ and $p < 0.004$).

Regional effects are reported with a voxelwise threshold of $p < 0.001$ (uncorrected); a individual cluster size correction of $p < 0.05$ was also adopted (see Table 1b). For this purpose we used an ad-hoc implementation of the methods described by Worsley [18,19] for non-isotropic images, as VBM data are. No regional effects outside those clusters are reported. We also indicate when a given region survived one of the two voxelwise corrections for multiple comparisons offered by SPM2, the Family-Wise Error correction (FWE) and the False Discovery Rate correction (FDR) [20].

## 3. Results

### *3.1. Neuropsychological results*

Descriptive statistics are reported in Table 1a. By design, there was a significant difference between the two groups of patients in the IR of the short story, while performance on the DR was at floor level in both groups. Using a Bonferroni correction, there was no other significant difference across groups of AD patients. However, there were substantial trends in two tests: the MMSE and the Raven Coloured Progressive Matrices (cRPM) test. An item-by-item analysis of the MMSE performance indicated that this trend was due to two subtests only (the "spatial orientation subtest" and the "sentence immediate repetition test") rather than to a generalized difference. Moreover, we did not find any significant correlation between MMSE and IR (S-AD = 0.276, G-AD = 0.182) or cRPM and IR in the two groups of patients.

Subject-by-subject analyses also indicated that among the G-AD patients there were 6 patients with preserved or close-to-normal performance in tasks that most likely depend on frontal/executive functions, such as the phonemic fluency task (6/6), the cRPM task (5/6), the attentional visual-search task (5/6) and the Trail-making A test (6/6). We also found that 5 out of 11 G-AD patients scored within the normal range in the Token test.

A similar pattern of results was found also in the S-AD group. In particular we found six out of the ten S-AD patients with a close to normal performance at the phonemic fluency task, seven patients with a performance within two standard deviations from the mean of the control group at the cRPM task and at the Trail-making A test. Finally nine patients out of ten had a close to normal performance at the visual search test.

### *3.2. Voxel-based morphometry results*

The comparison of the entire sample of AD patients with the normal controls showed a reduction of GM density of the temporal lobe bilaterally, the orbital part of the inferior frontal gyrus, the parietal lobe, the occipital regions bilaterally (in particular in the medial regions) and in the head of the two caudate nucleii (Fig. 1A).

However, when compared with the S-AD group and with the elderly controls, the G-AD patients showed a significantly larger atrophy of the left anterior hippocampus (Fig. 1B). Interestingly, a more posterior part of the left hippocampus was equally atrophic in the two groups of AD patients (see the green bar graph in Fig. 1A).

The distribution of the GM density of the anterior hippocampus in relation with the IR scores is illustrated with a linear regression scatter plot in Fig. 1C (see also Table 1C). Five out of the six patients with spared performance in tests dependent on executive/frontal functions had a GM density in the bottom range of the distribution (data points highlighted with red circles in Fig. 1C), indicating that GM density in this region can be greatly reduced, while executive functions are relatively preserved.

## 4. Discussion

This study provides new behavioural and anatomical evidence for the characterisation of the nature of dissociation in the immediate and delayed prose recall in patients with mild dementia of the Alzheimer type. Discussed in the light of the revised working memory (WM) model, our and others' behavioural evidence [3] suggests that the episodic buffer is not embedded within the central executive. Indeed, the joint impairment of IR and DR may not need a substantial impairment of executive functions, as in six out of the eleven G-AD subjects performance in the executive/frontal tasks was within the normal range, while performance at IR was at floor level. Our VBM data are also consistent with this view as the frontal lobes, widely considered as the anatomical substrates for executive functions [21], were not more affected in the patients with a complete IR deficit. On the other hand, our VBM data indicate that the left anterior hippocampus, the core region of damage in the G-ADs, may be a key anatomical substrate for the episodic buffer and for the encoding of bound memory traces. This suggestion is consistent

Table 1
Demographic data, performance at neuropsychological tests and neuroanatomical comparisons between AD patients and controls

| A. | S-AD | G-AD | Controls |
|---|---|---|---|
| Male/Female | 3/7 | 5/6 | 9/13 |
| Age | 77,5 (6,25) | 75,5 (7,92) | 71,5 (4,75) |
| Education (years) | 8,5 (4,45) | 6,3 (1,90) | 11 (4,36) |
| MMSE[12] | 22.2 (2.09) | 17.2 (2.92) | 28,1 (1,11) |
| Immediate prose recall[13] | **3** (**1.15**)° | **0.1** (**0.3**)° | 9,7 (2,94) |
| Delayed prose recall[13] | 0.1 (0.31) | 0.1 (0.42) | 12,2 (2,97) |
| Phonemic fluency[14] | 19.6 (8.30) | 18.2 (7.84) | 31 (7,63) |
| Semantic fluency[14] | 10.3 (2.66) | 8.4 (1.78) | 18,8 (3.02) |
| Token test[13] | 30.1 (3.19) | 28.3 (3.15) | 32.8 (1.46) |
| Visual search[13] | 37.7 (8.97) | 29.7 (10.62) | 46.8 (8.91) |
| Trail making test A[15] | 138,7 (54,2) | 196,2 (127) | 85,6 (47) |
| Digit span[16] | 5.2 (0.79) | 4.8 (0.98) | 5.7 (0.81) |
| Corsi's span[16] | 3.9 (0.31) | 3.7 (0.46) | 4.7 (0.7) |
| Raven's test[17] | 22.6 (5.14) | 15.8 (6.72) | 29.2 (3.71) |

| **B.** | x | y | z | Z score | x | y | z | Z score |
|---|---|---|---|---|---|---|---|---|
| **Brain regions** | **Left hemisphere** | | | | **Right hemisphere** | | | |
| Inferior frontal gyrus pars orbitalis | – | – | – | – | 47 | 18 | −11 | 4.1# |
| Inferior parietal lobule | −28 | −55 | 42 | 3.9# | – | – | – | – |
| Superior occipital gyrus | −26 | −71 | 41 | 4.3# | 27 | −72 | 37 | 3.6# |
| Superior temporal gyrus | −51 | −12 | 3 | 4.4 | 51 | −11 | −1 | 4.1# |
| Middle temporal gyrus | – | – | – | – | 57 | −39 | 2 | 4.2# |
| Inferior temporal gyrus | −48 | −36 | −16 | 5.0*# | 51 | −30 | −17 | 4.5# |
| Fusiform gyrus | −16 | 7 | −41 | 4.4# | 41 | −39 | −10 | 3.2# |
| Caudate | −7 | 19 | 1 | 3.8# | 6 | 12 | 2 | 3.4# |
| Anterior Hippocampus | −22 | −13 | −15 | 4.9*# | – | – | – | – |
| Middle Hippocampus | −34 | −25 | −14 | 4.5# | 39 | −24 | −11 | 4.9*# |

| **C.** | x | y | z | Z score | x | y | z | Z score |
|---|---|---|---|---|---|---|---|---|
| **Brain regions** | **Left hemisphere** | | | | **Right hemisphere** | | | |
| Anterior Hippocampus | −23 | −10 | −13 | 4.1** | – | – | – | – |

**A.** Descriptive statistics for the three groups (average and standard deviation). ° = significant difference between S-AD and G-AD (threshold for corrected statistical significance P-value: 0.0015.) **B.** Brain regions of grey matter density loss in G-ADs and S-ADs in comparison with elderly normal controls. **C**. Brain regions of grey matter density loss in G-AD patients in comparison with S-ADs and elderly normal controls. *= significant FWE correction $P < 0.05$ or more. # = significant FDR correction $P < 0.05$ or more. **= $p < 0.05$ FEW corrected (Z score:4.7) in a linear regression analysis between grey matter density and immediate recall scores.

with evidence coming from different levels of investigation, including in vitro recording in the hippocampus [22], observations in brain damaged patients [23] or functional imaging investigations in normal subjects (review in reference 24). In a summary of the vast literature of human imaging data related to episodic memory, Buckner et al. suggested the existence of a fronto-temporal network for information encoding, where the frontal cortex provides "the necessary ingredients" for the medial temporal lobe structures to bind together different information and form episodic memories [24]. This view is also supported by several more recent fMRI studies [25–29].

Our data are also in line with the recent suggestion of a functional specialization within the hippocampal formation which is not limited to its coronal axis, with the well known distinction between the dentate gyrus and the Ammon horn, but also to the antero-posterior axis, with the anterior part of the hippocampus being involved in associative memory tasks [30–32]. Our data provide further evidence in favour of this general proposal, as the most anterior part of the hippocampus appears crucial for the temporary maintenance of complex memory traces in working memory, while the more posterior hippocampal and para-hippocampal regions, affected in both patient groups, may be involved in longer-term storage of the information [33].

Finally, the present anatomical results are at variance with previous fMRI data based on paradigms that may be relevant for the EB [4–6]. In short, these studies suggest that the right PFC plays a role in the EB. However, it has to be considered that the experimental conditions and the materials used in those experiments might have not placed sufficient demands on the

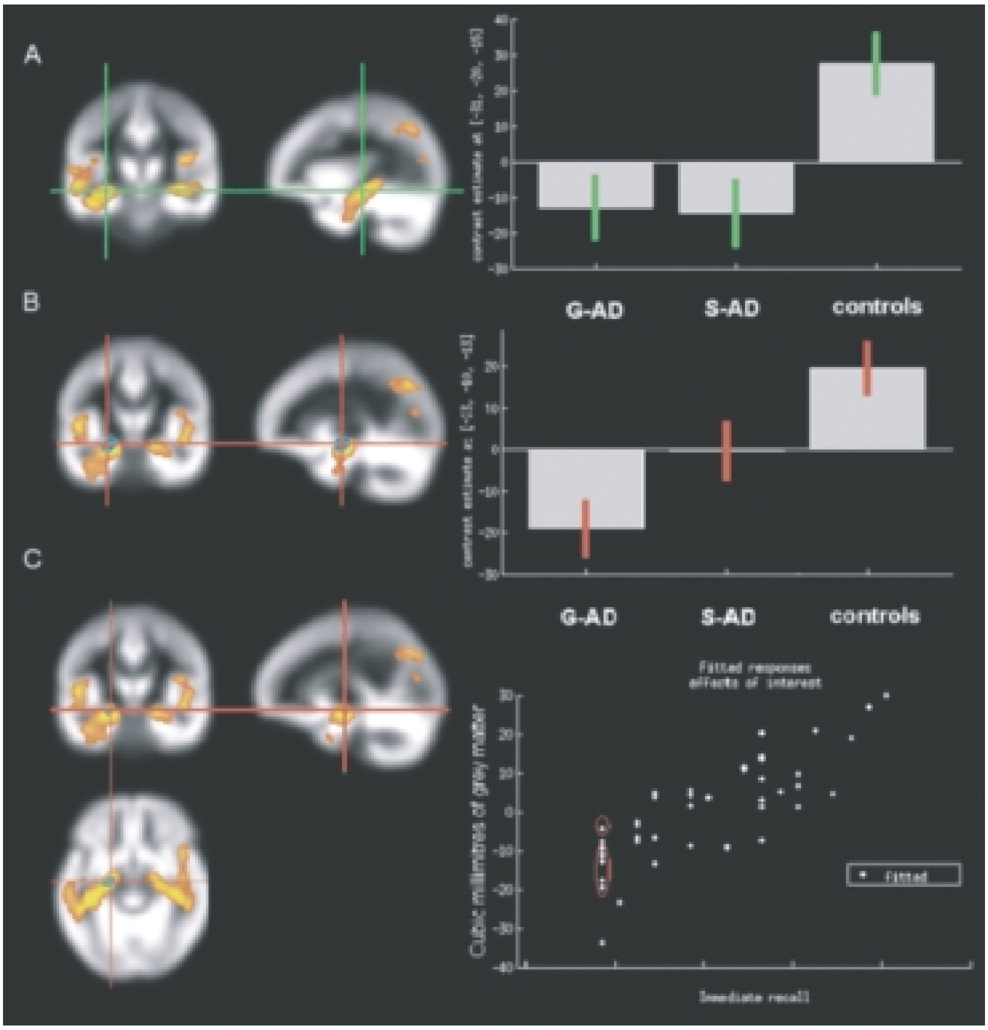

Fig. 1. A. Areas of grey matter density (GM) loss in the two AD patient groups compared with elderly controls. The green-cross indicates the posterior hippocampus in the Cornus Ammonis. The plot shows the level of mean GM density at the stereotactic coordinates $x = -31$, $y = -20$, $z = -15$. B. Brain regions of specific atrophy in G-ADs (in blue, centred by a red cross) in the anterior part of the hippocampus, in the Cornus Ammonis (stereotactic coordinates: $x = -23$, $y = -10$, $Z = -10$): the bar-plot shows the level of mean GM density in this area for the three groups. The brain regions in orange are regions of shared athrophy in the G-ADs and S-ADs. C. Correlation between GM density and performance at the immediate recall task in the anterior part of the hippocampus (stereotactic coordinates: $x = -21$, $y = -12$, $z = -13$; brain region in blue, centred by a red cross) across patients and normal controls. Red circles indicate data points of five G-AD patients with spared performance in tests dependent on executive/frontal functions. The brain regions in orange are regions of shared athrophy in the G-ADs and S-ADs.

hippocampal activity during the fMRI scans as stimuli could not be integrated at a semantic level or learned in long-term memory; moreover, those experimental tasks were very different from our task of interest (the IR of a short story with semantic content). Accordingly, we suggest that the results from those fMRI studies should be considered as complementary rather than in contradiction with our VBM data. Further studies, in which one manipulates the level of semantic information of the experimental stimuli to be held in the working memory, may contribute to clarify the discrepancy between fMRI and our VBM data.

## Acknowledgments

This study was supported by a grant from the Italian Ministry of Health and by PRIN 2005 to EP and GB, and by Bracco s.p.a. to G.B. We are grateful to Dr. Satoru Hayasaka for providing the cluster size correction software for VBM data.
